# Risk distribution of human infections with avian influenza A (H5N1, H5N6, H9N2 and H7N9) viruses in China

**DOI:** 10.3389/fpubh.2024.1448974

**Published:** 2024-10-24

**Authors:** Rongrong Qu, Mengsha Chen, Can Chen, Kexin Cao, Xiaoyue Wu, Wenkai Zhou, Jiaxing Qi, Jiani Miao, Dong Yan, Shigui Yang

**Affiliations:** ^1^Department of Emergency Medicine, Second Affiliated Hospital, Department of Epidemiology and Biostatistics, School of Public Health, The Key Laboratory of Intelligent Preventive Medicine of Zhejiang Province, Zhejiang University School of Medicine, Hangzhou, China; ^2^State Key Laboratory for Diagnosis and Treatment of Infectious Diseases, National Clinical Research Center for Infectious Diseases, National Medical Center for Infectious Diseases, Collaborative Innovation Center for Diagnosis and Treatment of Infectious Diseases, The First Affiliated Hospital, Zhejiang University School of Medicine, Hangzhou, China

**Keywords:** avian influenza A viruses (H5N1), H5N6, H9N2, H7N9, distribution, boosted regression tree model

## Abstract

**Background:**

This study aimed to investigate epidemiologic characteristics of major human infection with avian influenza and explore the factors underlying the spatial distributions, particularly H5N6 and H9N2, as H9N2 could directly infect mankind and contribute partial or even whole internal genes to generate novel human-lethal reassortants such as H5N6. They pose potential threats to public health and agriculture.

**Methods:**

This study collected cases of H5N1, H5N6, H9N2, and H7N9 in China, along with data on ecoclimatic, environmental, social and demographic factors at the provincial level. Boosted regression tree (BRT) models, a popular approach to ecological studies, has been commonly used for risk mapping of infectious diseases, therefore, it was used to investigate the association between these variables and the occurrence of human cases for each subtype, as well as to map the probabilities of human infections.

**Results:**

A total of 1,123 H5N1, H5N6, H9N2, and H7N9 human cases have been collected in China from 2011 to 2024. Factors including density of pig and density of human population emerged as common significant predictors for H5N1 (relative contributions: 5.3, 5.8%), H5N6 (10.8, 6.4%), H9N2 (11.2, 7.3%), and H7N9 (9.4, 8.0%) infection. Overall, each virus has its own ecological and social drivers. The predicted distribution probabilities for H5N1, H5N6, H9N2, and H7N9 presence are highest in Guangxi, Sichuan, Guangdong, and Jiangsu, respectively, with values of 0.86, 0.96, 0.93 and 0.99.

**Conclusion:**

This study highlighted the important role of social and demographic factors in the infection of different avian influenza, and suggested that monitoring and control of predicted high-risk areas should be prioritized.

## Introduction

Human infections with avian influenza viruses pose a persistent threat to public health. The first human infections with highly pathogenic avian influenza (HPAI) H5N1 virus occurred in Hong Kong in 1997, with 18 individuals infected, six of whom died (1). In 2013, China firstly detected cases of human infection with H7N9 viruses in Shanghai and experienced several waves of H7N9 epidemics (2). It was confirmed that the low pathogenic avian influenza H7N9 virus has evolved into HPAI viruses in February 2017, which may lead to wider spread and higher of risk for human or poultry (3).

H9N2 virus has spread more extensively, since it first isolated from turkeys in Wisconsin, USA in 1966 (4), causing constant infections in various countries. In 2021, H9N2 viruses were observed in Sub-Saharan Africa, historically considered a cold spot for animal influenza A virus (5). H9N2 virus could cause outbreaks in poultry, wild waterfowl and infect human directly despite of its low pathogenicity (6). It achieves cross host transmission through contributing partial or even whole set of internal protein functional segments to other subtypes of avian influenza viruses such as H5N1, H7N9, H10N8 and H5N6. In May 2014, the world’s first human infection with H5N6 virus was identified in Sichuan, China (7). H5N6 virus is also a highly pathogenic avian influenza virus and a reassortment virus, with both H5N1 and H9N2 viruses involved in the reassortment (8).

Countries around the world have reported cases of human infection with avian influenza virus through their national surveillance systems (9). Currently, few researches have explored what factors and how have contributed to the spatial distribution of human infections with avian influenza virus, especially H9N2 and H5N6. The aim of this study was to describe the characteristics and distribution of global human avian influenza cases, and explore the role of ecoclimatic, environmental, social and demographic factors which favor the occurrence of human infections in China, and thus to optimize resources allocated to controlling the disease and reducing the risk for human infection with avian influenza infection.

## Materials and methods

### Data on human cases with avian influenza virus infections

The data on human cases infected H5N1, H5N6, H9N2 and H7N9 from 2011 to 2024 were collected from the World Health Organization, including the WHO’s Disease Outbreak News of the Global Alert and Response (GAR), the WHO’s Weekly Epidemiological Record and the WHO Western Pacific Region’s Avian Influenza Weekly Update and ProMed-mail.^1^ Age, gender, outcome, symptom, the date of onset, the date of hospitalization, the date of death, location (country and province) of each confirmed patient, and poultry exposure was extracted and used in this study. The definitions of human infections with avian influenza viruses (H5N1, H5N6, H9N2 and H7N9) have been described according to WHO and previous studies (10–12).

### Data on ecoclimatic, environmental, social and demographic factors

A wide range of environmental and social factors that are commonly used in ecological studies on the spatial distribution of human infection with avian influenza virus were collected. The selection of factors was primarily based on empirical ecological evidence in published literature. The province-level ecoclimatic, environmental and social data were collected: 19 cross-sectional ecoclimatic variables (BIO01–19) (13), the density of human population, the number of live poultry markets, the density of pig, land cover variables including the percentage coverage of irrigation and wetland. In China, the provincial level is the key administrative unit to formulate public health policies and allocate health resources. In addition, data from provincial units are more complete and systematic, providing a relatively complete surveillance. Details on these variables and their estimates in the modeling analysis were described in Supplementary Table 1.

### Ecological modeling

For each of 4 major human infection with avian influenza subtypes in China reported by WHO, predictive machine learning models were developed at the provincial level using case–control study design. In brief, for each subtype, provinces with at least one reported case were recognized as “cases,” while those did not report any cases were considered as “controls.” BRT models is an efficient and popular approach for ecological studies to map infectious diseases such as tick-borne pathogens disease (14), anthrax (15), and helminth (16), identify risk determiners, and predict distributions of organisms, which has an obvious advantage is that allowing nonlinear covariate-outcome relationships and multicollinearity among covariates (14).

In this study, all provinces reporting cases were considered as “cases,” and five-fold “controls” were randomly chosen from the remaining provinces without reported cases for each model, and the case–control ratio is according to previous research (case–control ratio was 1: 5) to compose a balanced bootstrap set. For the establishment of BRT model, a bootstrapping procedure was used to provide a robust estimation of model parameters.

In BRT model, tree complexity determines the maximum split depth of each regression tree and thus controls the ability of the model to capture non-linear relationships. We chose a moderate tree complexity to ensure that the model could capture the interaction between features without resulting in over-fitting. The learning rate controls the weight of each tree’s contribution to the overall model prediction. A lower learning rate usually improves the predictive performance of the model, but more iterations are required to achieve optimal performance. We used a smaller learning rate (0.005) to ensure that each update had less impact on the final model, to avoid overfitting, and to improve the robustness of the model. Bagging fraction controls the proportion of training samples used to build the tree in each iteration. We selected an out-of-pocket sampling ratio of less than 1 (0.7) to introduce randomness, reduce the variance of the model, and improve the generalizability of the model. Therefore, a tree complexity of 4, a learning rate of 0.005 and a bagging fraction of 75% were used for the primary analysis to identify the optimal tree as previous studies (11, 17). A training set with 70% of the points were randomly selected from the current bootstrap data and the remaining 30% served as a test set.

The ROC curves and areas under the curve (AUC) based on the test sets and train sets were averaged separately to represent the final predictive performance. ROC curve is a common tool to evaluate the performance of classification model. It evaluates the discriminant ability of the model by showing the relationship between true positive rate and false positive rate under different decision thresholds. And AUC indicates the ability of the model to correctly distinguish between positive and negative samples, ranging from 0 to 1.

Finally, the mean value and standard deviation of estimated weights over 50 resampled datasets were reported. The relative contributions of included variables were estimated from the identified trees and served as an indicator of each variable’s importance for predicting human infection with H5N1, H5N6, H9N2 or H7N9 presence or absence. Variables that had a high contribution to the occurrence of the human infection avian influenza virus disease (weight > 2%) were included in the final model. BRT models also reported the predicted probabilities of occurrence of human infection. R packages dismo and gbm was to conduct models, and predictive power was evaluated using pROC (R v4.3.3 environment).

## Results

### Spatial and temporal distribution of avian influenza

A total of 1,123 H5N1, H5N6, H7N9, and H9N2 human cases are collected from January 2011 to March 2024 in China. The total number of H7N9 cases was greater than other subtypes (Table 1). The male-to-female ratio varied across subtypes, with a higher proportion of males for H5N1 and H7N9. The human infection with different subtypes also shows a very different age-specific epidemiology. The median age of H5N1 cases was 38.5 years, with an inter quartile range (IQR) from 20 to 49.3 years. The median age of H5N6 and H7N9 cases is similar (52 years and 58 years). And H9N2 cases is the youngest (median and IQR: 5 and 2–9 years). The proportion of recovery is greater than deaths for all subtypes, and most of the cases had history of poultry exposure. Compared to other subtypes, H7N9 cases are more likely to be severe. Median time from illness onset to hospital admission of H5N1 cases was longer than other subtypes. For H7N9 cases, the median time from hospital admission to death was longer than for other subtypes. Median time from illness onset to death of H5N6 cases was longer than other subtypes.

**Table 1:** The characteristics of human case with H5N1, H5N6, H7N9, and H9N2 virus infection, January 2011 to March 2024.

Categories	H5N1 (*n* = 32)	H5N6 (*n* = 133)	H7N9 (*n* = 826)	H9N2 (*n* = 132)
Age, year (Median, IQR)	38.5 (20–49.3), *n* = 28	52 (37–58), *n* = 129	58 (46–67), *n* = 815	5 (2–9), *n* = 126
Male (*n*, %)	19 (67.9), *n* = 28	60 (45.8), *n* = 131	576 (70.8), *n* = 814	45 (37.5), *n* = 120
Outcome (*n*, %)				
Died	5, 15.6	29, 21.8	136, 16.5	-
Recovered	27, 84.4	104, 78.2	690, 83.5	132, 100
Poultry exposure (*n*, %)				
Any exposure to poultry	14, 43.8	62, 46.6	101, 12.2	44, 33.3
Visited live poultry market	4, 12.5	22, 16.5	286, 34.6	19, 14.4
Exposure to backyard poultry	2, 6.3	21, 15.8	119, 14.4	22, 16.7
Exposure to sick or dead poultry	1, 3.1	7, 5.3	1, 0.1	0, 0
Occupational exposure to live poultry	2, 6.3	0, 0	61, 44.2	0, 0
Contact with human cases of infection	0, 0	0, 0	10, 1.2	0, 0
No exposure or have unclear history of exposure	9, 28.1	21, 15.8	248, 30.0	47, 39.2
Disease severity (*n*, %)				
Mild	23, 71.9	80, 60.6	349, 42.3	130, 98.5
Severe	4, 12.5	24, 18.2	341, 41.3	2, 1.5
Fatal	5, 15.6	29, 21.8	136, 16.5	–
Median time from illness onset to hospital admission, d (IQR)	5 (3–7)	4 (1–5)	3 (1–3)	1 (0–6)
Median time from hospital admission to death, d (IQR)	2 (2–2)	9 (4.5–13.3)	10 (7–13)	–
Median time from illness onset to death, d (IQR)	5 (5–7)	13 (8.3–15.8)	11 (8–14)	–

**Figure 1:**
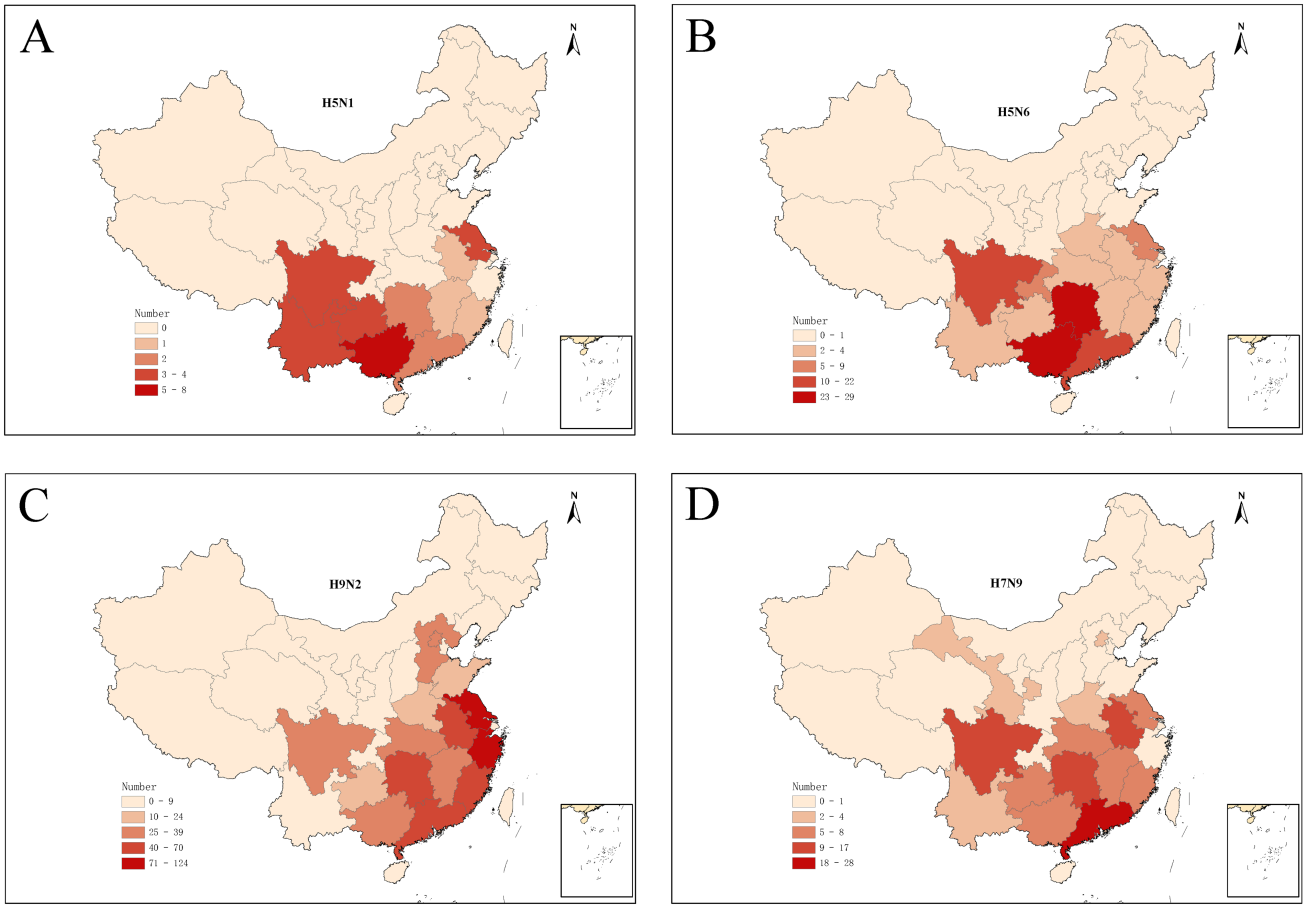
Spatial distribution of human infections with H5N1 **(A)**, H5N6 **(B)**, H9N2 **(C)**, and H7N9 **(D)** in China from January 2011 to March 2024.

**Figure 2:**
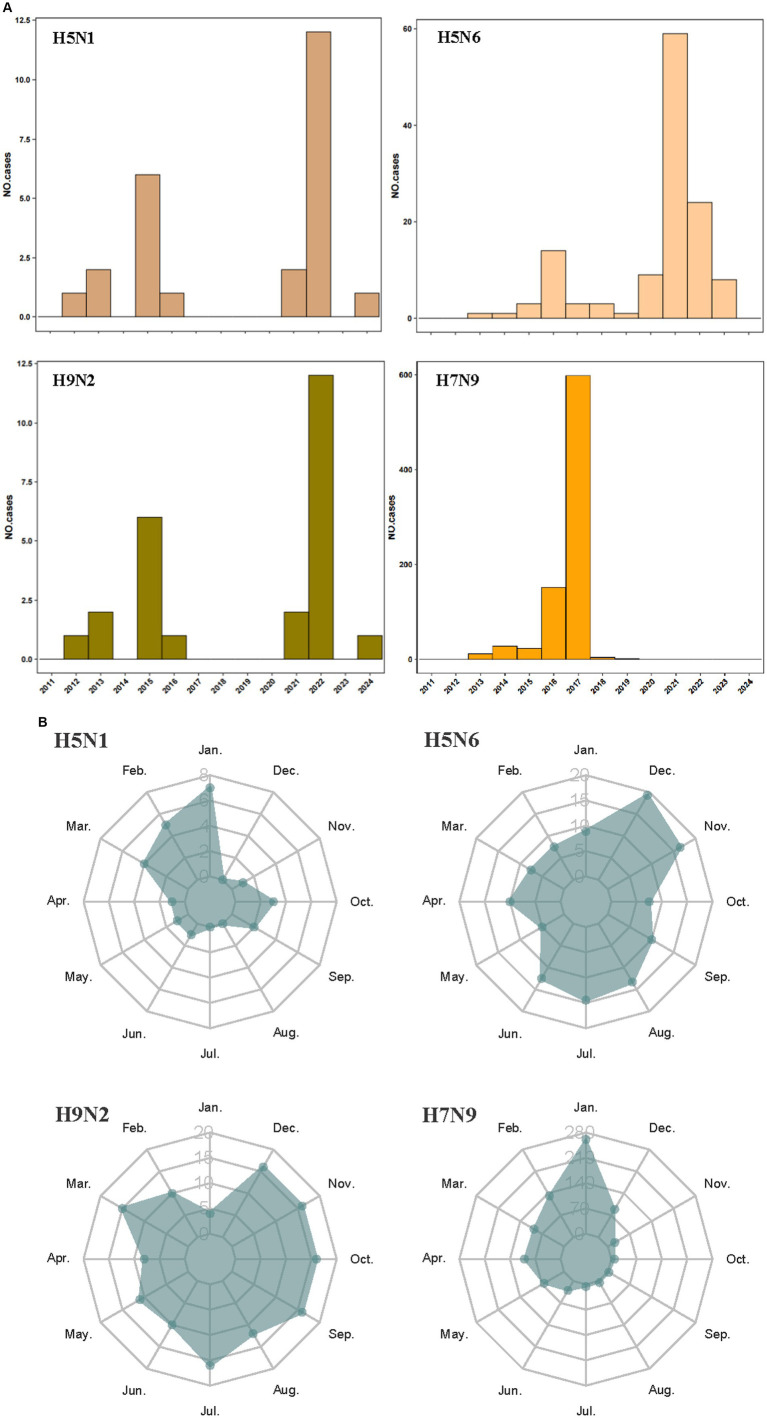
Temporal distribution of human infections with H5N1, H5N6, H9N2, and H7N9 in China from January 2011 to March 2024. Distribution by years of avian influenza cases **(A)**; distribution by season of avian influenza cases **(B)**.

The human infections with avian influenza virus (H5N1, H5N6, H7N9, and H9N2) from 2011 to 2024 displayed distinct spatial distribution in China (Figure 1). It exhibited the spatial distribution of human H5N1 cases in 11 provinces, H5N6 cases in 15 provinces and one municipality in China, H9N2 cases in 15 provinces and one municipality, and the distribution of human H7N9 cases in 26 provinces and four municipality. Intuitively, H5N1 and H5N6 cases are mainly concentrated in Eastern and Southwest China, while H9N2 and H7N9 infection is more widely distributed even penetrates into the northwest China. The annual number of human cases also shows significant variations (Figure 2A). The largest number of H5N1 and H9N2 cases were reported in 2022, with 12 and 36 patients, respectively, and the largest number of H5N6 cases was reported in the 2021, with a total of 59 patients, while the majority of H7N9 cases were reported in 2017, with a total of 598 patients. Human H7N9 and H5N1 infections exhibited seasonality, with more cases occurring in the cool/cold season, while there are sporadic H5N6 and H9N2 cases were reported throughout the year (Figure 2B).

### Risk factors for avian influenza

Further, the results revealed that, for all 4 viruses infection, a higher risk of human infection was associated with density of pig and density of human population (relative contributions >5%) (Table 2). In addition, the relative contribution of the number of live poultry markets, precipitation of warmest quarter, precipitation of wettest quarter, percentage coverage of wetland, mean temperature of coldest quarter, and precipitation of wettest month for H5N1 are 15.4% (−2.04–32.84%), 15.0% (1.28–28.72%), 14.7% (−2.55–31.9%), 13.8% (−3.45–31.05%), 8.4% (−4.928–21.728%), and 7.1% (−3.68–17.88%). The occurrence of human infection with H5N6 were found to be significantly associated with the number of live poultry markets, the percentage coverage of irrigation, density of pig, precipitation of wettest quarter, precipitation of coldest quarter, with relative contributions of 42.9% (33.10–52.70%), 14.4% (9.90–18.91%), 10.8% (7.08–14.52%),8.9% (0.08–17.72%), and 5.3% (2.16%-8.44). The number of live poultry markets (relative contribution 22.3%) was found to be the most important variable in predicting the risk of human infection with H9N2 in the model, followed by percentage coverage of wetland (17.5%), the percentage coverage of irrigation (15.6%), mean temperature of warmest quarter (11.5%), precipitation of wettest quarter (9.5%), and mean temperature of wettest quarter (5.3%). In particular, the percentage coverage of irrigation played important roles in the occurrence of human infection with H7N9 (BRT mean weights are 68.5%), followed by density of pig (9.4%) and density of human population (8.0%).

**Table 2:** Results of the boosted regression trees applied to the occurrence of human infection with avian influenza A (H5N1, H5N6, H9N2 and H7N9) virus data.

Relative contribution												
	Human H5N1 infection			Human H5N6 infection			Human H9N2 infection			Human H7N9 infection		
Variable	Mean (%)	SD	95%CI	Mean (%)	SD	95%CI	Mean (%)	SD	95%CI	Mean (%)	SD	95%CI
LMP	15.4	8.9	−2.04 to 32.84	42.9	5.0	33.10–52.70	22.3	5.4	11.72–32.88	4.6	1.7	1.27–7.93
Irrigate	4.5	1.8	0.97–8.03	14.4	2.3	9.90–18.91	15.6	2.3	11.09–20.11	68.5	2.2	64.19–72.81
BIO19	–*	–	–	5.3	1.6	2.16–8.44	–	–	–	–	–	–
BIO18	15.0	7.0	1.28–28.72	2.1	1.9	−1.62–5.82	–	–	–	3.2	0.8	1.63–4.77
Density of pig	5.3	3.5	−1.56 to 12.16	10.8	1.9	7.08–14.52	11.2	2.1	7.08–15.32	9.4	1.1	7.24–11.56
BIO16	14.7	8.8	−2.55 to 31.95	8.9	4.5	0.08–17.72	9.5	4.1	1.46–17.54	–	–	–
Density of human population	5.8	2.4	1.10–10.50	6.4	1.3	3.85–8.95	7.3	1.7	3.97–10.63	8.0	3.8	0.55–15.45
BIO2	2.7	1.0	0.74–4.66	3.2	1.1	1.04–5.36	–	–	–	–	–	–
BIO10	–	–	–	–	–	–	11.5	6.5	−1.24–24.24	–	–	–
Wetland	13.8	8.8	−3.45 to 31.05	–	–	–	17.5	9.4	−0.92–35.92	–	–	–
BIO4	2.4	2.4	−2.304 to 7.104	–	–	–	–	–	–	–	–	
BIO7	2.8	2.3	−1.708 to 7.308	–	–	–	–	–		–	–	
BIO8	2.5	1.4	−0.244 to 5.244	–	–		5.3	2.1		–	–	
BIO9	3.8	3.7	−3.452 to 11.052	–	–		–	–		–	–	
BIO11	8.4	6.8	−4.928 to 21.728	–	–		–	–		–	–	
BIO12	2.0	1.2	−0.352 to 4.352	–	–		–	–		–	–	
BIO13	7.1	5.5	−3.68 to 17.88	–	–		2.1	0.8		–	–	
BIO14	2.5	1.7	−0.832 to 5.832	–	–		–	–		–	–	

*: “–” These variables were excluded from the final model due to small BRT weights (<2.0%). Variables with mean weights≥5% were considered as significant contributors to the occurrence of human infections. LMP, the number of live poultry markets; Irrigate, the percentage coverage of irrigation; BIO02, mean diurnal range; BIO04, temperature seasonality; BIO07, annual range of temperature; BIO08, mean temperature of wettest quarter; BIO09, mean temperature of driest quarter; BIO10, mean temperature of warmest quarter; BIO11, mean temperature of coldest quarter; BIO12, annual precipitation; BIO13, precipitation of wettest month; BIO14, precipitation of driest month; BIO16, precipitation of wettest quarter; BIO18, precipitation of warmest quarter; BIO19, precipitation of coldest quarter.

**Figure 3:**
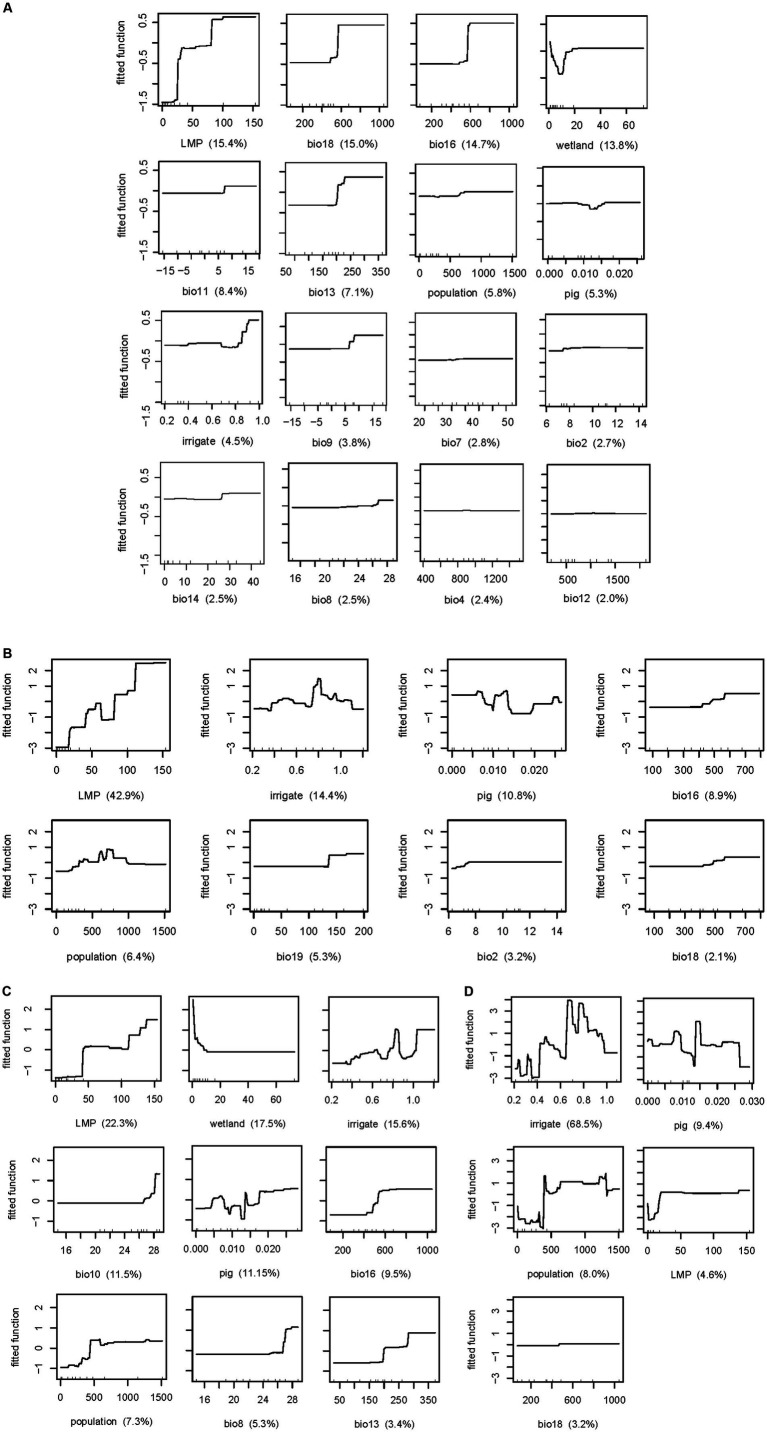
Relationship between risk factors and human infection with avian influenza H5N1 **(A)**, H5N6 **(B)**, H9N2 **(C)**, H7N9 **(D)** risk. LMP, the number of live poultry markets; irrigate, the percentage coverage of irrigation; population, density of human population; pig, density of pig; wetland, the percentage coverage of wetland. BIO02, mean diurnal range; BIO04, temperature seasonality; BIO07, annual range of temperature; BIO08, mean temperature of wettest quarter; BIO09, mean temperature of driest quarter; BIO10, mean temperature of warmest quarter; BIO11, mean temperature of coldest quarter; BIO12, annual precipitation; BIO13, precipitation of wettest month; BIO14, precipitation of driest month; BIO16, precipitation of wettest quarter; BIO18, precipitation of warmest quarter; BIO19, precipitation of coldest quarter.

Although the four viruses share several variables as common risk factors, the effect patterns differ among them. The fitted functions plotted based on the BRT model showed that the occurrence of human H5N1 infection increased with the number of live poultry markets, precipitation of warmest quarter, precipitation of wettest quarter, mean temperature of coldest quarter, and precipitation of wettest month, whereas the percentage coverage of wetland manifested an opposite trend (relative contributions >5%) (Figure 3A). Specifically, the risk associated with live poultry markets clearly escalates once the number of such markets reaches 30, and it increases even further when this number reaches 80, highlighting the importance of regulating and monitoring such markets to control virus spread. Similar risk patterns are observed in relation to the density of the human population, precipitation levels in the warmest and wettest quarters, mean temperature during the coldest quarter, and precipitation in the wettest month. In these cases, the risk intensifies swiftly after surpassing a specific threshold before stabilizing. These results emphasizes the need for targeted surveillance and intervention efforts during high-risk periods, such as the wettest and warmest months. Furthermore, the inverse relationship with the percentage coverage of wetland might point to natural ecosystem services provided by wetlands that reduce the likelihood of human exposure to the virus, perhaps by maintaining ecological balance and reducing interactions between wild birds and humans. Therefore, preserving wetlands could serve as an indirect but valuable public health strategy.

The fitted functions for human infection with H5N6, as depicted based on the BRT model, indicated that the occurrence of human infection increased with the number of live poultry markets, precipitation of the wettest quarter, and precipitation of the coldest quarter (Figure 3B). The risk of the number of live poultry markets exhibited a stepwise increase, stabilizing after reaching 110, underscoring the critical need for stricter biosecurity measures and regulation in areas with a high concentration of such markets. Policies aimed at reducing market density might be recommended to mitigate infection risk. When the precipitation of the wettest quarter is below 400 mm, the risk is minimal. It then gradually increases as the precipitation levels rise, reaching a stabilization point after surpassing 600 mm. Similarly, lower risk is observed when the precipitation of coldest quarter is lower than 130 mm, with risk escalating as precipitation levels rise, peaking at 135 mm before stabilizing. These implied that surveillance efforts should be intensified during periods of heavy rainfall. However, the effect of the percentage coverage of irrigation, density of pig, and density of human population on the risk of H5N6 infection appears to be non-monotone (Figure 3B), indicating a more complex relationship between these variables and the risk of infection, which may be associated with local ecological and socio-economic contexts.

For human infection with H9N2 viruses, the number of live poultry markets, mean temperature of warmest quarter, precipitation of wettest quarter, density of human population, and mean temperature of wettest quarter share a similar risk pattern with increasing trend, while the percentage coverage of wetland showed a totally opposite trend (Figure 3C). Specifically, when the number of live poultry markets exceeds 40, the risk increases significantly. The mean temperature of warmest quarter exceeds 27°C, the risk increases evidently; similarly, mean temperature of wettest quarter exceeds 27°C, the risk also increases evidently. And precipitation of wettest quarter reaches 400 mm, the risk elevated. In addition, density of human population, reaches 500 person per km^2^, the risk was higher than those lower 500 person per km^2^. The risk is high when the percentage coverage of wetland is lower than 10% and dropped thereafter. The percentage coverage of irrigation and density of pig also shows non-monotone risk curve, with the peak risk reached at 85 and 14%, respectively.

In contrast, there are fewer risk factors for H7N9. Generally, a higher the percentage coverage of irrigation, density of pig, and density of human population were associated with a higher risk of H7N9 infection, also in a non-monotone manner, with the peak risk reached at 70, 15%, and 1,250 person per km^2^, respectively (Figure 3D). The risk is high when the number of live poultry markets is more than 20, although the relative contributionsis only 4.6%, lower than 5%, which also indicated its role in H7N9 infection. Overall, each virus has its own ecological and social drivers for human infection. The number of live poultry markets and precipitation of warmest quarter contributed the most to H5N1 infection. For H5N6 infection, the key factors were precipitation of wettest quarter and the number of live poultry markets. H9N2 infection was most influenced by the number of live poultry markets and the percentage coverage of wetlands, while the percentage coverage of irrigation and pig density were the most significant contributors to H7N9 infection.

**Figure 4:**
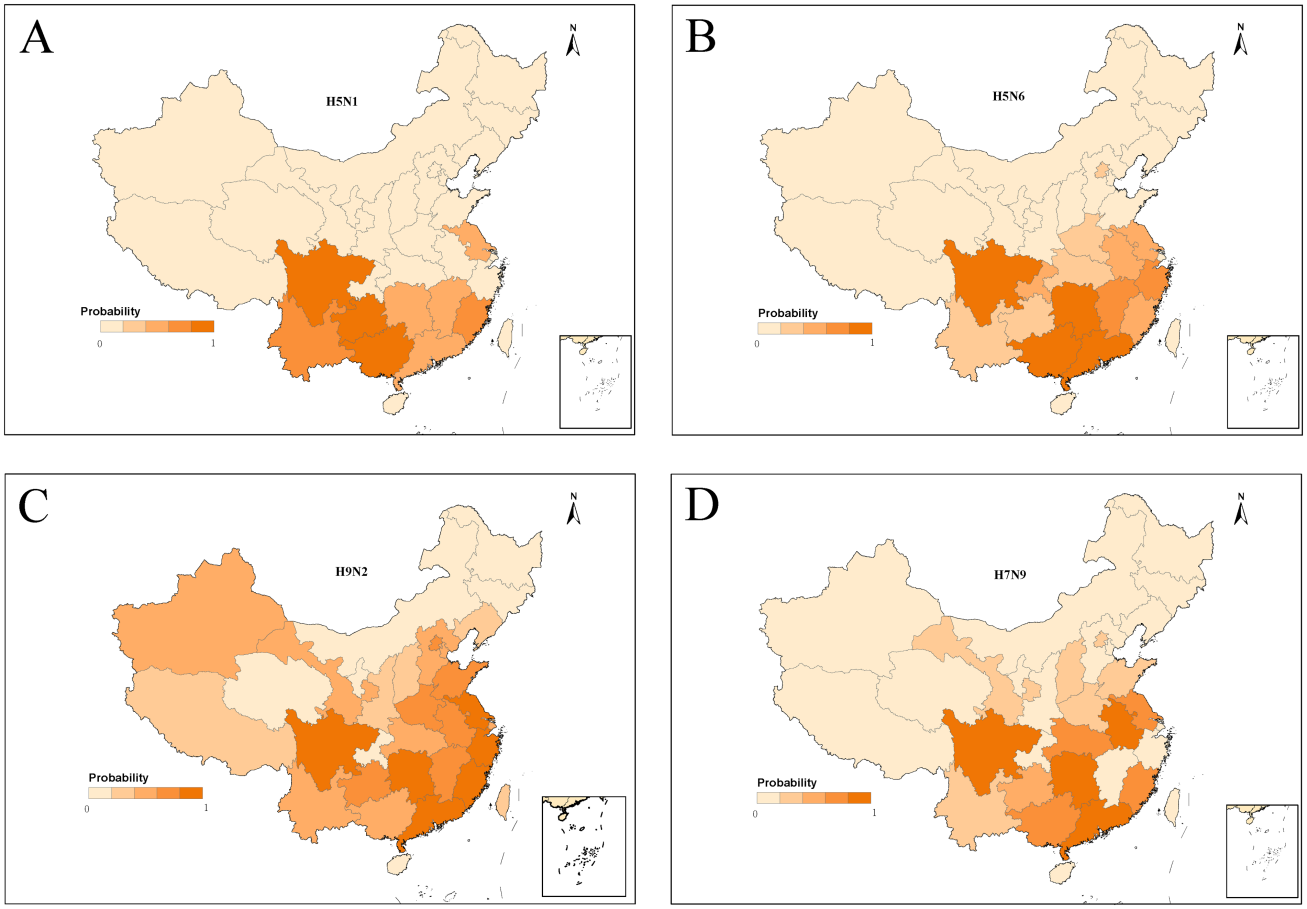
Predictive risk maps of probability of occurrence of human infections with H5N1 **(A)**, H5N6 **(B)**, H9N2 **(C)**, and H7N9 **(D)** in China, and darker red indicating a higher risk.

To acquire the model-fitted probability of occurrence of human infection with H5N1, H5N6, H9N2, and H7N9, a bootstrapping procedure for the BRT model was performed to create robust estimates. The spatial distribution of the model-predicted risk areas of occurrence of human infections was demonstrated in Figure 4. The distributions of model-predicted risk resembled the regions where are currently collected. The predicted distribution probabilities for H5N1 presence in southwestern China including Guangxi, Sichuan, Guizhou, and Yunnan is very high, with values of 0.86, 0.82, 0.80 and 0.60, respectively. The predicted map of H5N6 showed that the highest risk areas were southeastern China, extending from the Pearl River delta near Guangzhou to the Yangtze River delta near Shanghai and covering most areas of Jiangsu, Zhejiang, Anhui, Fujian, Jiangxi, Hubei, Guizhou, Yunnan, Henan Provinces, even Beijing. Notably, hot spots for human infections with H5N6 were found in Sichuan, Hunan, Guangdong Province and Guangxi Autonomous Region. The distributions of high-risk areas of H9N2 were similar with H5N6. Compared to H5N6 and H9N2, the high-risk areas of H7N9 are more geographically extensive, and might occur even in the most northwestern part of China such as Xinjiang and Inner Mongolia Autonomous Region.

To evaluate the discriminatory power, the receiver-operating characteristic (ROC) curve was plotted for the BRT model and area under the curve (AUC) was calculated. The accuracy metrics of the predictions produced by the BRT models are excellent, with mean AUC values estimated using the evaluation dataset ranging from 0.986 to 0.997 (Supplementary Figure 1). As expected, the AUC estimated based on the training data is always better than that estimated using the evaluation dataset.

## Discussion

The cases in China were collected from January 2011 to March 2024. Individual-level characteristics vary between subtypes as previous study (18). The spatial distribution of H5N1, H5N6, H9N2, and H7N9 indicated some differences. The density of pig and density of human population were identified potential warning factors for the incidence of H5N1, H5N6, H9N2 and H7N9 subtype infections. Furthermore, the distributions of BRT model-predicted risk are similar with the areas where are currently collected. To the best of our knowledge, this is the first study to predict the geographic distribution of human infection with avian influenza A (H5N6) and (H9N2) by boosted regression tree model in China.

Prior to the emergence of the highly pathogenic H7N9 avian influenza at the end of December 2016, the subtype had mild or even no symptoms in poultry, making surveillance difficult. But the highly pathogenic strain of H7N9 virus was found in 2017, spreading rapidly through poultry in the following months. As for H5N6, the number of reported cases suddenly increased significantly in 2021, and 4 new genotypes detected in 2021 were the major causes of increased H5N6 virus infections (19).

Previous studies have indicated that environmental factors also affect the spread of avian influenza, such temperature and relative humidity (20). In addition, BRT model has been employed in predicting the potential geographic distribution of H7N9 and H5N1, identifying suitable areas for disease occurrence and assessing risk factors. Martin et al. found that HPAI H5N1 clinical disease outbreak occurrence in domestic poultry was primarily linked to chicken density, human population density, and elevation (17). Fang et al. discovered that live poultry markets, human population density, irrigated croplands, built-up land, relative humidity and temperature significantly contributed to the occurrence of human infection with H7N9 virus (11). In addition, Li et al. reported that live poultry markets, density of human, coverage of built-up land, relative humidity and precipitation were significant predictors for both viruses (21). As noted by previous studies, live poultry markets serve as critical places for avian influenza transmission due to the high density of animal–animal and human-animal interactions. The present results reinforced critical role of the number of live poultry markets in human infection risk across multiple avian influenza viruses. The consistent findings likely stem from the fact that these markets provide ideal conditions for virus transmission, including direct contact between humans and infected birds, as well as the potential for virus persistence in contaminated environments.

In the present study, it is interesting to note that human infections with H5N1, H5N6, H9N2 and H7N9 share similar social demographic factors (density of pig and density of human population). High density of human population and density of pig lead to a high risk of H5N1, H5N6, H9N2 and H7N9 infections. One interpretation is that densely populated areas are more likely to be associated with poultry-related trading or farming, which may promote the spread of pathogens in animal hosts and increase the chances of human infection with avian influenza. Another possible reason is that patients are more likely to be observed in areas with more people and timely medical facilities. When the population density reaches a certain threshold, the risk of infection tends to level out, which may be explained by virus’s adaption to man. The ability of a virus to spread from one species to another is determined by many factors such as the availability of an intermediate host, most commonly pigs, which plays a genetic mixing vessel role between birds and humans (22, 23). Therefore, the important role of the density of pig for the four subtypes is not surprising.

The relationship between occurrence of human cases and the percentage coverage of irrigation suggests that waterfowl would act as hosts in the transmission of avian influenza viruses. It has been known that domestic ducks could excrete large numbers of virus through salivary and nasal secretions and feces although seems to be healthy (24), thus water is considered as a mediator to facilitate the transmission of viruses from waterfowl to human without direct contacts. Avian influenza virus has been tested to remain infective for up to 207 days at 17°C and up to 102 days at 28°C (25), which increases the probability of human infection. From a historical perspective, China has developed agriculture from the need to feed the people as efficiently as possible, using limited resources. Domestic ducks were first migrated from rivers to cultivated rice paddies in the mid-17th century during the early Qing Dynasty (26). This practice helps to protect the growing rice from pests and reduces usage of chemical insecticides. However, it provides a closer proximity for ducks, water, food and people.

The live poultry market has always been recognized as the reservoir and amplifier of avian influenza viruses, where highly crowded environments and frequent poultry exposure are common (27–29). In the present study, live poultry markets had a lower influence on the occurrence of human H7N9 infection than other subtypes infection based on BRT model. This difference might due to the different transmissibilities from animal hosts to human across viruses but requires further investigation.

Human infection with H5N1 and H9N2 was both affected by the percentage coverage of wetland. As mentioned above, domestic ducks moved from natural rivers to farmland, the number of important hosts in wetland decreased between viruses and human, which may partly explain the negative association between occurrence of H5N1, H9N2 and the percentage coverage of wetland. In the context of continuing the implementation of “return of farmland to lakes” policies, attention should be paid to the ecological impact of the migration of wild birds in wetlands.

Precipitation is the principal climatic variables contributing to human infections for H5N1, H5N6, H9N2 and H7N9 viruses, while mean temperature of warmest quarter only affected the human H9N2 infections risk. Similar with previous results of the association between H5N1 infection and relative humidity (21), BRT models showed that higher precipitation created a higher risk of H5N1 and H5N6. Higher precipitation always indicates the rainy day. In that case, people prefer to stay indoors and increase the physically person-to-person and animal-to-person distance. Consistent with the finding of precipitation, a higher mean temperature of warmest quarter and mean temperature of wettest quarter (after 26°C) was associated with a higher risk of H5N1 and H9N2 infection. Global ecosystem is susceptible to impacts from changing climate (30). Current evidence suggests that groundwater and lake temperatures have been increasing over the past 40 years, causing water temperature variation up to 3°C. Warming temperatures are considered significant for wildlife disease dynamics and may lead to increased avian influenza virus pathogenicity or transmissibility through influencing bird migration patterns or other ways (31).

Furthermore, the risk maps provided valuable reference for decision-makers and public health officials. For example, the Pearl River and Yangtze River deltas, identified as having a high probability of H5N6 infection, which was consistent with previous studies (32), should be prioritized for enhanced surveillance, stricter biosecurity measures in live poultry markets. In areas like southwestern China, where H5N1 infection risks are elevated, public health authorities can implement targeted vaccination programs for poultry, monitor potential outbreaks more closely, and ensure rapid response capabilities. Notably, the identification of hot spots for multiple virus strains, such as in Guangxi, Sichuan, and Guangdong, suggests that these regions may require multi-faceted strategies to address the risks of different avian influenza viruses. Understanding these spatial patterns facilitates more efficient deployment of diagnostic resources, the establishment of quarantine zones if necessary, and the timely dissemination of health advice to high-risk populations. The maps can also be used by policymakers to assess the need for cross-provincial coordination and international collaboration, especially in areas near provincial borders or regions with significant poultry trade.

The advantage of this study was that BRT model was used to examine the association between ecoclimatic, environmental, social and demographic factors and several predominant human infections with avian influenza from 2011 to 2024 in China. This study also has some limitations. First, the sample of human infection with avian influenza viruses was collected from reporting system. It is possible that many mild cases have already occurred but were not detected due to the incomplete coverage of the sentinel surveillance network in several less developed areas of China or not seeking medical care, and thus are subject to reporting bias. Second, the relatively smaller cases of human H5N6 and H9N2 infections reported in China may limit the discriminating power of the BRT models, as demonstrated by the lower AUCs compared to H7N9. Third, live poultry movements with broiler and layer poultry supply chains (such as breeding, hatching, slaughter, wholesale and retail markets) are considered to be too complex to be described on provincial scales (33). So, the number of live poultry markets included in the BRT model is not precise. In addition, human cases infected with four subtypes were collected the provincial level in China during 2011–2024, which may be crude scale. And other environmental and socio-economic factors that could influence infection risk such as elevation, coverage of built-up land and public awareness were not included in the analysis. Finally, it should be noted that the BRT model was employed to predict the probability of occurrence of human infection with avian influenza instead of making causal inferences.

## Conclusion

In conclusion, targeted interventions such as continuous monitoring of pig density and population density is necessary. In addition, mapping predictive risk of various avian influenza viruses based on models offers a valuable approach to identify the areas where surveillance efforts should be targeted.

## Data Availability

The original contributions presented in the study are included in the article/Supplementary material, further inquiries can be directed to the corresponding authors.

## Footnotes

^1^ https://promedmail.org/
