# Supplementary material for: Risk distribution of human infections with avian influenza A (H5N1, H5N6, H9N2 and H7N9) viruses in China
Source: Front Public Health. 2024 Oct 24;12:1448974. doi: 10.3389/fpubh.2024.1448974 (PMC11540643; doi:10.3389/fpubh.2024.1448974)
Supplement: Supplementary file 1 [file Table_1.docx]

Supplementary Table 1. The ecoclimatic, environmental and social variables used for ecological modeling for human infection with H5N1, H5N6, H9N2 and H7N9 in this study.

| Category | Variable | Description | Resource |
| --- | --- | --- | --- |
| Ecoclimatic^1^ | BIO01 | Annual mean temperature (℃) | Worldclimate  https://worldclim.org/ |
|  | BIO02 | Mean diurnal range (Mean of monthly (max temp-min temp)) (℃) |  |
|  | BIO03 | Isothermality (BIO02/BIO07)(*100) |  |
|  | BIO04 | Temperature seasonality (standard deviation*100) |  |
|  | BIO05 | Max temperature of warmest month (℃) |  |
|  | BIO06 | Min temperature of coldest month (℃) |  |
|  | BIO07 | Annual range of temperature (BIO05-BIO06) (℃) |  |
|  | BIO08 | Mean temperature of wettest quarter (℃) |  |
|  | BIO09 | Mean temperature of driest quarter (℃) |  |
|  | BIO10 | Mean temperature of warmest quarter (℃) |  |
|  | BIO11 | Mean temperature of coldest quarter (℃) |  |
|  | BIO12 | Annual precipitation (mm) |  |
|  | BIO13 | Precipitation of wettest month (mm) |  |
|  | BIO14 | Precipitation of driest month (mm) |  |
|  | BIO15 | Precipitation seasonality(Coefficient of variation) |  |
|  | BIO16 | Precipitation of wettest quarter (mm) |  |
|  | BIO17 | Precipitation of driest quarter (mm) |  |
|  | BIO18 | Precipitation of warmest quarter (mm) |  |
|  | BIO19 | Precipitation of coldest quarter (mm) |  |
| Demographics | the density of human population^2^ | The number of population / total area | the National Bureau of Statistics of China |
| Social | the density of pig^3^ | The number of pig/ total area | the National Bureau of Statistics of China |
|  | LMP^3^ | The number of live poultry markets | www.autonavi.com |
| Environmental^4^ | Wetland | Percentage coverage of wetland (%) | the National Bureau of Statistics of China |
|  | Irrigation | Percentage coverage of irrigation land (%) | the National Bureau of Statistics of China |

^1^These ecoclimatic variables perform better than traditional meteorological variables (such as mean temperature and relative humidity) in reflecting seasonal trends of different species associated with their physiological constraints, thus having been extensively applied in ecological research[1]. ^2^Several anthropogenic factors were observed to be linked to avian influenza in a lot of studies performed in countries varying in agro-ecological and production conditions such as Thailand and Vietnam [2] Therefore, the density of human population was included. The number of population of each province and area of each province were obtained from the National Bureau of Statistics of China. ^3^The poultry-related variables such as number of live poultry markets and the density of pigs are potential predictors for the exposure level of human hosts to the potential sources of infection. ^4^The land cover variables could reflect to some extent the habitats and food sources of wild birds and poultry[3]. Data regarding the density of pigs, land cover variables including the percentage coverage of irrigation and wetland were also derived from the National Bureau of Statistics of China. The number of live poultry markets was obtained from AutoNavi, a Chinese location based service (www.autonavi.com) as previously[4].


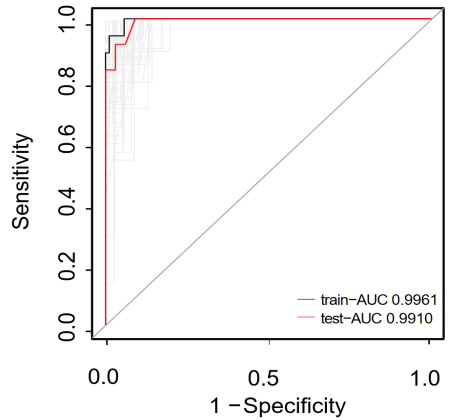


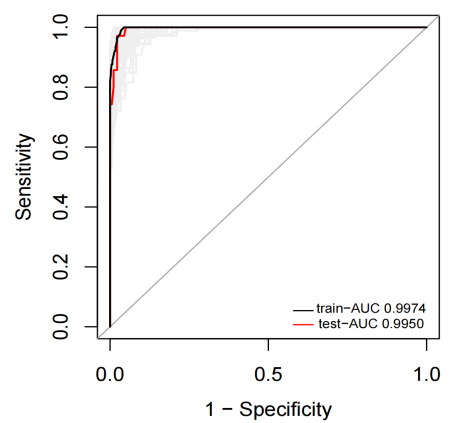


H5N6

H9N2

H7N9


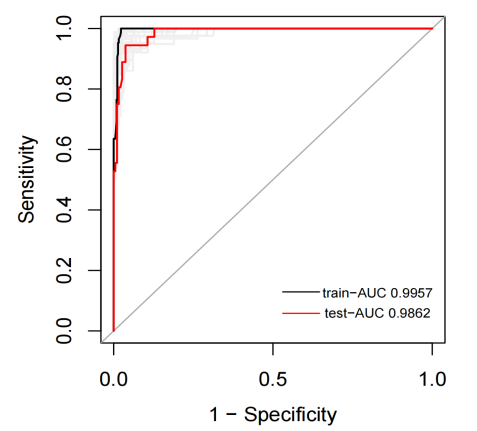

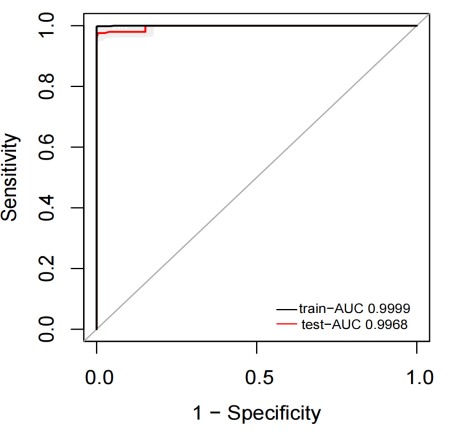


H5N1

**Fig S1**. ROC curves of the predicted risk of H5N1, H5N6, H9N2 or H7N9 presence/absence.ROC curves for BRT models: the grey lines are the ROC curve for each repeat, and the black and red lines indicate the average ROC curves of 50 repeats based on the bootstrapping procedure for the train set and test set.

**Reference**

1. Schurz H, Salie M, Tromp G, Hoal EG, Kinnear CJ, Möller M: **The X chromosome and sex-specific effects in infectious disease susceptibility**. *Hum Genomics* 2019, **13**(1):2.

2. Gilbert M, Xiao X, Pfeiffer DU, Epprecht M, Boles S, Czarnecki C, Chaitaweesub P, Kalpravidh W, Minh PQ, Otte MJ *et al*: **Mapping H5N1 highly pathogenic avian influenza risk in Southeast Asia**. *Proc Natl Acad Sci U S A* 2008, **105**(12):4769-4774.

3. Li XL, Yang Y, Sun Y, Chen WJ, Sun RX, Liu K, Ma MJ, Liang S, Yao HW, Gray GC *et al*: **Risk Distribution of Human Infections with Avian Influenza H7N9 and H5N1 virus in China**. *Sci Rep* 2015, **5**:18610.

4. Fang LQ, Li XL, Liu K, Li YJ, Yao HW, Liang S, Yang Y, Feng ZJ, Gray GC, Cao WC: **Mapping spread and risk of avian influenza A (H7N9) in China**. *Sci Rep* 2013, **3**:2722.
